# Short-term high-fat diet favors the appearances of apoptosis and gliosis by activation of ERK1/2/p38MAPK pathways in brain

**DOI:** 10.18632/aging.203607

**Published:** 2021-10-07

**Authors:** Chao-Jin Xu, Mei-Qi Li, Li-Zhao, Wei-Guang Chen, Jun-Ling Wang

**Affiliations:** 1 Department of Histology and Embryology, School of Basic Medical Science, Wenzhou Medical University, Wenzhou, Zhejiang 325035, PR China; 2 School of 2nd Clinical Medical Sciences, Wenzhou Medical University, Wenzhou, Zhejiang 325035, PR China; 3 Center for Reproductive Medicine, Affiliated Hospital 1 of Wenzhou Medical University, Wenzhou, Zhejiang 325000, PR China

**Keywords:** apoptosis, cerebral cortex, cerebellum, ERK1/2, high-fat, p38MAPK

## Abstract

High-fat diet (HFD) has been associated with neuroinflammation and apoptosis in distinct brain regions. To explore the effect of short-term (7, 14 and 21 days) high-fat overfeeding on apoptosis, inflammatory signaling proteins, APP changes and glial cell activities in cerebral cortex and cerebellum. Mice were fed with HFD for different lengths (up to 21 days) and after each time body weights of mice was tested, then the apoptotic proteins, IL-1β, APP, BACE1and MAPKs, Akt and NF-κB signaling activity were evaluated by western blots. Results demonstrate that short period of high-fat overnutrition significantly promotes apoptosis, APP expression at day 21 of cerebral cortex and at day 7 of cerebellum compared to chow diet. In addition, increased GFAP^+^astrocytes, Iba-1^+^microglia and IL-1β 30 were observed in cerebral cortex after 21 days HFD, but no changes for 7 days overfeeding of cerebellum. Serendipitously, ERK1/2 pathway was activated both in cerebral cortex and cerebellum for different time course of HFD. Furthermore, increased phospho-p38 MAPK level was observed in cerebellum only. In consistent with *in vivo* results, SH-SY5Y cells treatment with cholesterol (50 μM, 100 μM) for 48 h culture *in vitro* demonstrated that pro-apoptotic proteins were enhanced as well. In brief, short-term HFD consumption increases sensitivity to apoptosis, APP and IL-1β production as well as gliosis in cerebral cortex and cerebellum, which may be related to enhancement of ERK1/2 and p38 MAPK activation.

## INTRODUCTION

The worldwide prevalence of obesity is increasing worldwide and the main cause of obesity is intake of a high-fat diet (HFD), also known as the western diet [1]. Long-term consumption of HFD (≥30% of energy from fat) is associated with a pattern of chronic inflammation characterized by releasing pro-inflammatory cytokine and cytotoxic mediators in central nervous system (CNS) [2–4]. In addition, as major immune cell types in CNS, microglia and astrocytes are critical in regulating neuroinflammation and liberating inflammatory mediators [5]. Recent studies have suggested that HFD-associated neuroinflammation can become an important risk factor for the development of neurodegenerative disorders, such as Alzheimer’s disease (AD), and lead to cognitive deficits. Furthermore, a larger body of literature demonstrate that prolonged HFD-induced cognitive impairment exhibit increased apoptosis in the hippocampal neurons [6–10]. These studies have suggested that HFD intake increases susceptibility of hippocampus to excessive proinflammatory responses.

Interestingly, even as little as 3–10 days exposure, short-term HFD consumption has demonstrated that production of proinflammatory cytokines such as interleukin-1β (IL-1β) and tumor necrosis factor α (TNFα), apoptosis and memory impairments in humans and rodents in long before obesity symptoms appearance [11–15]. Nevertheless, a literature suggests that short-term HFD overnutrition does not induce NF-κB inflammatory signaling of subcutaneous white adipose tissue in humans [16].

On the other hand, mounting evidence has indicated that mitogen-activated protein kinase (MAPK) pathway is involved in the cellular responses to the metabolic stresses like HFD [17–20]. MAPK pathways, consisting of ERK1/2, ERK5, JNK, and p38/SAPKs, play complex space– and time–dependent patterns and participate in cell survival and apoptosis [21]. For example, enhanced ERK signaling contributes to cellular survival in short time, but the delayed activation of this kinase can lead to cell death [22]. In addition, while p38/MAPK signaling has been implicated in initiating apoptosis, p38 MAPK can also sometimes prevent cell death under some conditions [23]. However, less abundant evidence is available showing whether short-term HFD intake could affect apoptosis, APP expression, inflammatory biomarkers and gliosis in cerebral cortex and cerebellum mediated by MAPKs signaling transduction. Therefore, the current study aimed to explore the potential effects of short-term HFD consumption (7, 14 and 21 days) on apoptosis proteins, IL-1β, APP (amyloid-beta precursor protein) production and activation of glial cell in different brain regions. We also investigate whether stress response pathways, such as MAPK signaling, are involved these process.

## MATERIALS AND METHODS

### Animals, diet compositions and treatments

Adult male C57BL/6J wild type mice (8-weeks of age) were purchased from Gem Phar ma tech Co., Ltd (Licensed production number: SCXK-(SU)-2018-0008; Nanjing, China). Low fat and no sugar Chow diet (TP23100) and High fat diet (HFD, TP23103) were purchased from Trophic Animal Feed High-tech Co., Ltd (Nantong, China). The Diet compositions were displayed in Table 1. After one week adaptation, mice were randomly divided into three groups (*n* = 5 mice/group). Control group mice were treated with chow diet. In addition, 7 days group, 14 days group and 21 days groups were fed with HFD for 7 days, 14 days and 21 days, respectively, Mice were subsequently humanely sacrificed. Brain specimens were collected and preserved at −80°C until further use. All animal experimental procedures were reviewed and approved by the Committee Guide of Wenzhou Medical University (ethical number 2019-75; Wenzhou, China.). All mice were maintained on a 12 hours light-dark cycle and temperature 24°C–25°C with free access to water and food. All surgery was conducted under sodium pentobarbital anesthesia and efforts were made to reduce animal suffering.

**Table 1:** Compositions of chow diet and high fat diet nutritional facts.

	**Chow Diet (g/100 g)**	**High Fat Diet (g/100 g)**
Casein		24
Corn Starch		7.3
Maltodextrin		12
Sucrose		20.3
Soybean Oil		3
Lard		19.6
Cellulose		6
Mineral Mix, M1020		5.9
Vitamin Mix, V1010		1.2
L-Cystine		0.4
Choline Bitartrate		0.3
TBHQ		0.0045
Cholesterol		0.0141
	**Chow Diet (Kcal %)**	**High Fat Diet (Kcal %)**
Protein	26%	19.40%
Carbohydrate	64%	35.60%
Fat	10%	45%

### SH-SY5Y cell line culture and cholesterol treatment

SH-SY5Y Cells (ATCC) was were maintained in Dulbecco’s modified Eagle’s medium134 (HyClone, SH30022.01B) supplemented with 4 mM L-Glutamine (Sigma) and 10% fetal bovine serum (Gibco^®^), Cell lines was cultured at 37°C and 5% CO_2_. Cells were treated with 50 μM and 100 μM cholesterol for 48 h, respectively. Methods of protein extraction and immunoblotting analysis are showed in Immunoblotting analysis.

### Immunofluorescence (IF) staining

The immunofluorescent assay were carried out according to procedures performed by us [24, 25]. Briefly, brains were placed in fresh 30% sucrose solution for 24 h, then embedded in OCT compound (Sakura Finetek, Torrance, CA, USA) and cut at a thickness of 40 μm on a cryostat (CM1950; Leica, Mannheim, Germany). Brain sections were then incubated with following antibody for 1 h in room temperature: glial fibrillary acidic protein (GFAP; 1:400), a marker of astrocytes and ionized calcium binding adaptor molecule 1 (Iba-1; 1:400), a marker of microglia/macrophage-specific calcium-binding protein. After washing 30 min with PBS, then sections were incubated with Dy Light 488-conjugated goat anti-rabbit and goat anti-mouse (1:400; Jackson Immuno Research Labs) antibody for 1 h in room temperature, and washed three times with PBS. Stained sections were captured under a confocal laser scanning microscopy (Nikon Corporation, Tokyo, Japan). Optical density (OD) of immunoreactive structures were measured using ImageJ software (developed at the National Institutes of Health), similar method as previously have described by us [24].

### Immunoblotting analysis

The western blotting methods were performed according to guidelines previously established by our group [24, 26, 27]. In brief, mice were anesthetized with an intraperitoneal injection of pentobarbital sodium (50 mg/kg, i.p.) at day 7, day 14 and day 21 after high fat and chow diet feeding. Mice were then decapitated following brain extraction (cerebral cortex and cerebellum). The brain tissue were cut into smaller pieces and incubated in RIPA buffer (Beyotime Biotechnology; P0013B) through ultrasonic cell disruptor. After centrifugation, the supernatants were obtained. Proteins (10~15 μg) from lysates were separated on 5~10% Tris–glycine SDS–PAGE gels and transferred onto nitrocellulose membranes. Membranes were incubated at room temperature for 1 h in 5% (w/v) nonfat dry milk in TBST (Tris-buffered saline, 0.1% Tween 20), washed in TBST and incubated with primary antibodies overnight at 4°C. The primary antibodies were listed in Table 2. After incubation at 4°C, membranes were rinsed for 3 times (10 min each time) at room temperature with agitation in TBST, incubated with the species appropriate secondary antibody for 60 min at room temperature, and rinsed the membrane for a further 3 times (10 min each time) in TBST at room temperature before ECL detections. ImageJ software (National Institutes of Health) was used for quantification on densitometry of protein bands.

**Table 2:** Key resource table.

**Regent**	**Source**	**Identifier**
β-actin antibody	Affinity Biosciences	AF7018
EphA4 antibody	Affinity Biosciences	AF5496
His-Tag Mouse Monoclonal Antibody	Affinity Biosciences	T0009
NF-κB p65 antibody	Sigma Aldrich	SAB4502609
GADPH antibody	Sigma Aldrich	G8795
Caspase 8 antibody	Proteintech Group	66093-1-Ig
α–tubulin antibody	Cell Signaling Technology	#2144
ERK1/2 antibody	Cell Signaling Technology	137F5
Phospho-p44/42 MAPK	Cell Signaling Technology	4370S
A563	BD Biosciences	No. 612238
A620	gift from Dr. Wei-Lin, Jin	
Protein A/G PLUS-Agarose	Santa Cruz	sc-2003
Phospho-p44/42 MAPK (Erk1/2)	Cell Signaling Technology	4370S
Phospho-SAPK/JNK	Cell Signaling Technology	9255S
SAPK/JNK Antibody	Cell Signaling Technology	9252S
p38MAPK Antibody	Cell Signaling Technology	9212S
Phospho-p38 MAPK	Cell Signaling Technology	4511S
PARP (46D11) Rabbit mAb	Cell Signaling Technology	9532S
p53 (1C12) antibody	Cell Signaling Technology	#2524S
GFAP antibody	Cell Signaling Technology	#80788
Caspase-3 Antibody	Cell Signaling Technology	#9662
HRP-conjugated goat anti-mouse IgG	Beyotime Biotechnology	A0216
HRP-conjugated goat anti-rabbit IgG	Beyotime Biotechnology	A0208
PageRuler™ Plus Prestained Protein Ladder	No: 26619	Thermo Fisher
Dual Color Prestained Protein Marker	Epizyme Biotechnology	No: WJ102
Cell lysis buffer	Beyotime Biotechnology	P0013
Coomassie Blue Fast Staining Solution	Beyotime Biotechnology	P0017
IPTG	Beyotime Biotechnology	ST098-5g
kanamycin	Beyotime Biotechnology	ST102
Ni-NTA agarose	Qiagen Inc., Valencia	
*In situ* apoptosis detection (TUNEL) kit	Roche, Switzerland	
Dehydrocorydaline chloride	MedChem Express company	HY-N0674A
SP600125	MedChem Express company	HY-12041
Rhynchophylline (Rhy)	Baoji Herbest Bio-Tech	

SH-SY5Y Cells were rinsed twice with PBS and lysed in RIPA buffer for 30 min on ice. Protein collecting form SH-SY5Y Cells and western blotting also used the aforementioned procedure.

### Statistical analysis

Experiments were repeated at triple or four times independently. All data were reported as Mean ± SD (standard deviation). All statistical analyses were performed using GraphPad software (GraphPad Prism version 8.00, San Diego, CA, USA). The body weight data of the mouse were analyzed by two-way ANOVA followed by Sidak’s multiple comparisons test to compare differences between both groups. The additional data with three or more groups were analyzed with a one-way ANOVA, followed by post hoc testing with Tukey LSD. Differences between two groups were tested by unpaired two-tailed Student’s *t*-test. The significance level were set when ^*^P < 0.05, highly as significant when ^**^P < 0.01.

### Availability of data and materials

All data generated or analyzed during this study are included in this published article.

## RESULTS

### Effects of short-term HFD on total body weight, expression of amyloid precursor protein (APP) and BACE1 in a mouse model at two months of age

**Figure 1:**
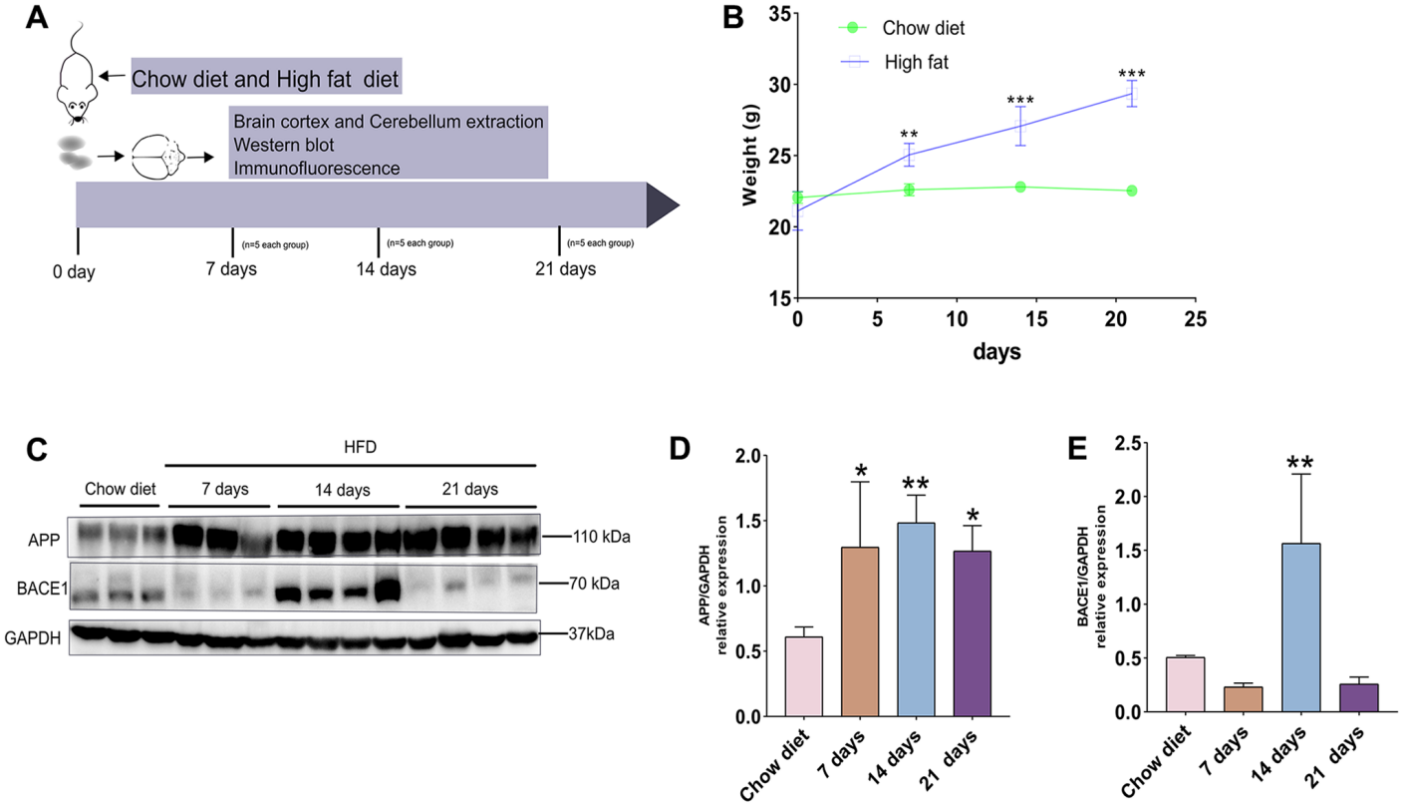
**A timeline exhibiting expression changes of APP and BACE1 after short-term HFD exposure for mice cerebral cortex.** (**A**) Experimental scheme for testing the effect of short period HFD on brain of adult mice. (**B**) Body weight curve of the two treatment groups at different time courses. (**C**) Western blotting and (**D**–**E**) densitometry analysis for APP and BACE1 from cerebral cortex tissue lysates obtained from mice with chow diet and HFD (*n* = 3 to 4 per group). GAPDH, glyceraldehyde-3-phosphate dehydrogenase. Diet compositions are shown in Table 1. Data shown as means ± SD. ^*^P < 0.05, ^**^P < 0.01, and ^***^P < 0.001 vs. chow diet group, by two-way ANOVA followed by Bonferroni’s post hoc test for body weight analysis and one-way ANOVA by Tukey’s test for densitometry assay.

To identify whether short-term intake of HFD could significantly raise body mass, mice from the two diet groups were weighed on the day of diet beginning (day 0) and tested every seven days (Figure 1A). As illustrated in Figure 1B, the body weight of HFD-fed mice were significantly raised compared to that of chow diet mice on day 0 (22.06 ± 0.33 g vs. 21.12 ± 1.10 g; *p* < 0.01; *n* = 5 per group), on day 7 (22.60 ±0.34 g vs. 25.06 ± 0.65 g; *p* < 0.01; *n* = 5 per group), on day 14 (22.8 ± 0.29 g vs. 27.08 ± 1.11 g; *p* < 0.001; *n* = 5 per group), on day 21 (22.54 ± 0.22 g vs. 29.36 ± 0.75 g; *p* < 0.001; *n* = 5 per group).

By western blotting, we next analyzed APP production and expression of BACE1 (a key enzyme involved in APP metabolism) at different time-point (Figure 1C). Short-term HFD intake elevated the level of APP after 7 days, 14 days and 21 days, compared to chow diet groups (*p* < 0.05; *p* < 0.01) (Figure 1D). On the other hand, BACE1 expression were significantly up-regulated at day 14 after HFD exposure, but this protein reduced to normal level compared to chow diet groups after 21 days HFD (*p* < 0.01) (Figure 1E). Thus, our results demonstrates that increased protein expression of APP is achieved by responding to short-term HFD. In addition, fluctuations of BACE1 proteins during short-term HFD demonstrate APP metabolism mediated by BACE1 is complex *in vivo.*

### Effects of short-term HFD on apoptosis proteins and aging marker p21 in cerebral cortex

**Figure 2:**
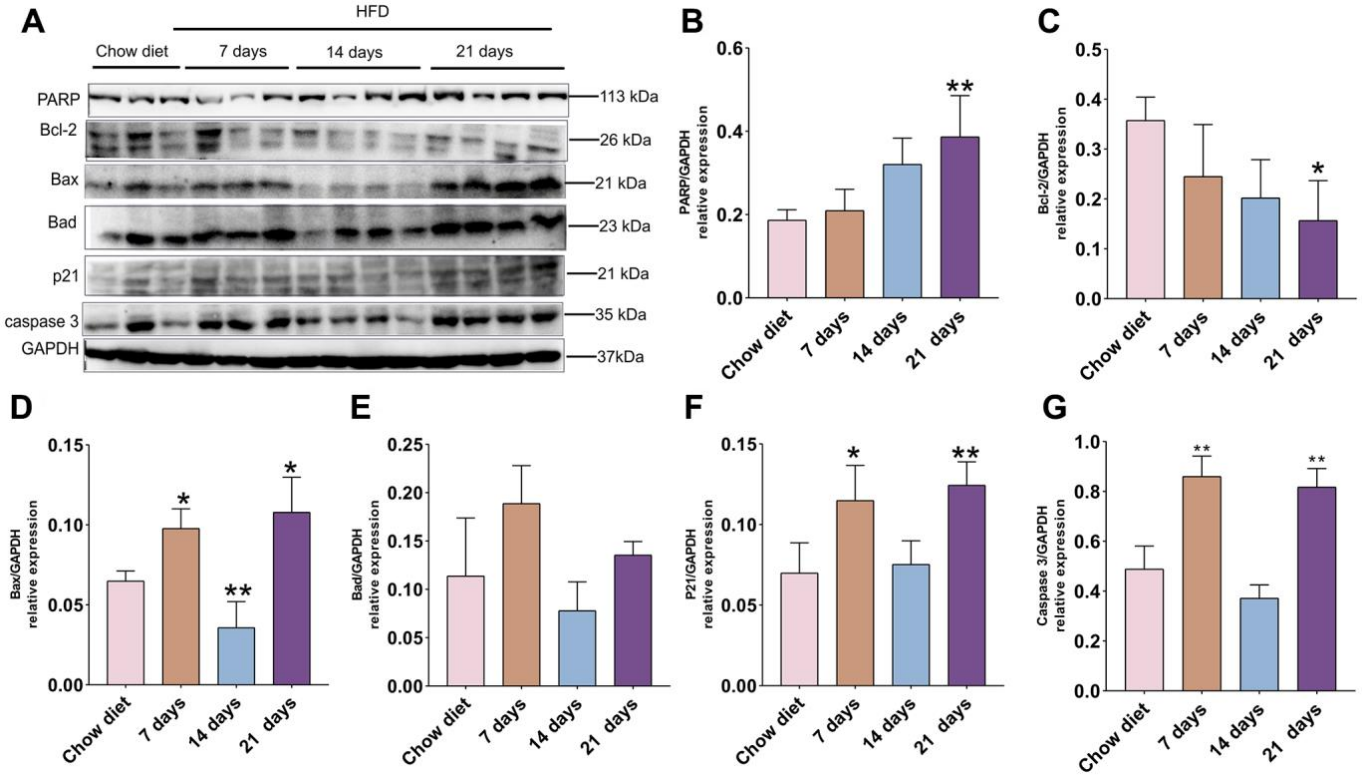
**Association of apoptosis and HFD in mice cerebral cortex at separate times.** (**A**) Western blot analysis of cerebral cortex tissues of mice with HFD and chow diet controls (*n* = 3 to 4 per group). (**B**–**G**) Quantification of PARP (**B**), Bcl-2 (**C**), Bax (**D**), Bad (**E**), p21 (**F**), caspase3 (**G**). GAPDH as a loading control. Values are presented as means ± SD. ^*^P < 0.05 and ^**^P < 0.01 versus chow diet; one-way ANOVA by Tukey’s test.

As demonstrated in Figure 2A, the PARP protein level was significantly enhanced at day 21 compared to chow diet groups (*p* < 0.01) (Figure 2B). In contrast to PARP expression, the anti-apoptotic BCL-2 protein showed opposite trend and significantly reduced after 21 days HFD compared to chow diet mice (*p* < 0.05) (Figure 2C). The pro-apoptotic Bax was raised at day 7 and 21 and reduced at day 14 compared to chow diet groups (*p* < 0.05; *p* < 0.01) (Figure 2D). Intriguingly, no significant difference changes were found for pro-apoptotic Bad at different time-points (Figure 2E). Furthermore, cerebral cortex senescence was significantly induced at day 7 and 21 compared to chow diet (*p* < 0.05; *p* < 0.01) (Figure 2F) and confirmed by p21, which invokes cell cycle arrest and senescence [28]. On the other hand, caspase-3 protein exhibited similar results as the p21 protein expression (*p* < 0.01) (Figure 2G). Taken together, these findings indicate that short-term consumption of a HFD not only accelerates cell death through apoptosis but also advances senescence in cerebral cortex.

### Changes of APP, BACE1, apoptosis proteins in cerebellum after 7 days HFD

**Figure 3:**
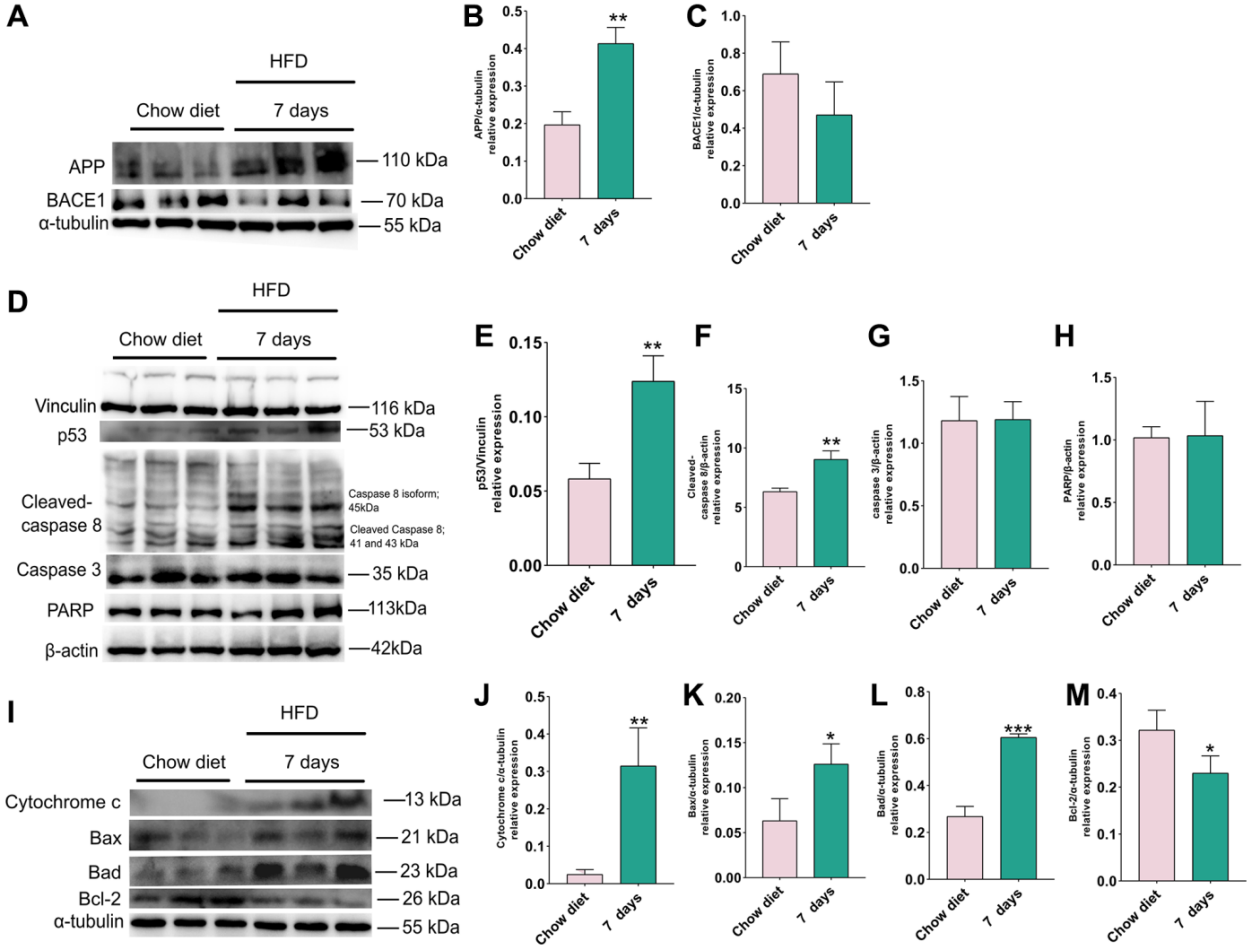
**Changes of APP, BACE1 and apoptosis proteins after 7 days of HFD in the mice cerebellum.** (**A**) Western blots and (**B**–**C**) densitometric analysis for APP, BACE1 in the supernatants of mice cerebellum (*n* = 3). (**D**) Western blot and (**E**–**H**) densitometric quantification of p53 (**E**), cleaved-caspase8 (**F**), caspase 3 (**G**), PARP (**H**) indicated (*n* = 3). (**I**) Western blot analysis of apoptotic proteins in cerebellum tissue lysates of indicated day 7 after HFD exposure for mice (*n* = 3). (**J**–**M**) Quantification of cytochrome C protein (**J**), Bax (**K**), Bad (**L**), Bcl-2 (**M**). Vinculin, β-actin and α-tubulin as a loading control. Values are means ± SD. ^*^P < 0.05 and ^**^P < 0.01 versus chow diet; by two-tailed Student’s *t* test.

To evaluate how 7 days of HFD feeding affects the activities of APP, BACE1 and cell death, cerebellum proteins were analyzed by western blotting. Similarly, the results indicated that level of APP was also significantly elevated at day 7 compared to chow diet (*p* < 0.01) (Figure 3A–3B), however, BACE1 did not demonstrate obvious changes (Figure 3C).

In the next step, we investigated whether 7 days HFD consumption also change the apoptotic signal in cerebellum, western blots were used to assess the changes of p53, cleaved-caspase 8, caspase 3, PARP, cytochrome C, Bax, Bad and BCL-2 (Figure 3). Statistical analysis shows that the amounts of p53 (Figure 3D, 3E) and cleaved-caspase 8 (Figure 3D, 3F) were evidently enhanced after consumption of HFD for 7 days (*p* < 0.01), however, this short period of HFD did not bring about significant difference in cerebellum caspase 3 and PARP activity as illustrated in Figure 3G and 3H.

Similar results were also found for apoptotic proteins released from the mitochondria. As depicted in Figure 3I, statistical analysis demonstrated that pro-apoptotic proteins cytochrome C, Bax, Bad were increased significantly (*p* < 0.01; *p* < 0.05) (Figure 3J–3L), however, in this time point (7 days) a significant reduction in in cerebellum anti-apoptotic BCL-2 activity could be detected (*p* < 0.05) (Figure 3M). Together, the results demonstrate that intake of 7 days HFD elevate the level of APP and accelerate the occurrence of apoptosis in cerebellum.

### Effects of short-term HFD on IL-1β production, glial cell activation markers in cerebral cortex and cerebellum

**Figure 4:**
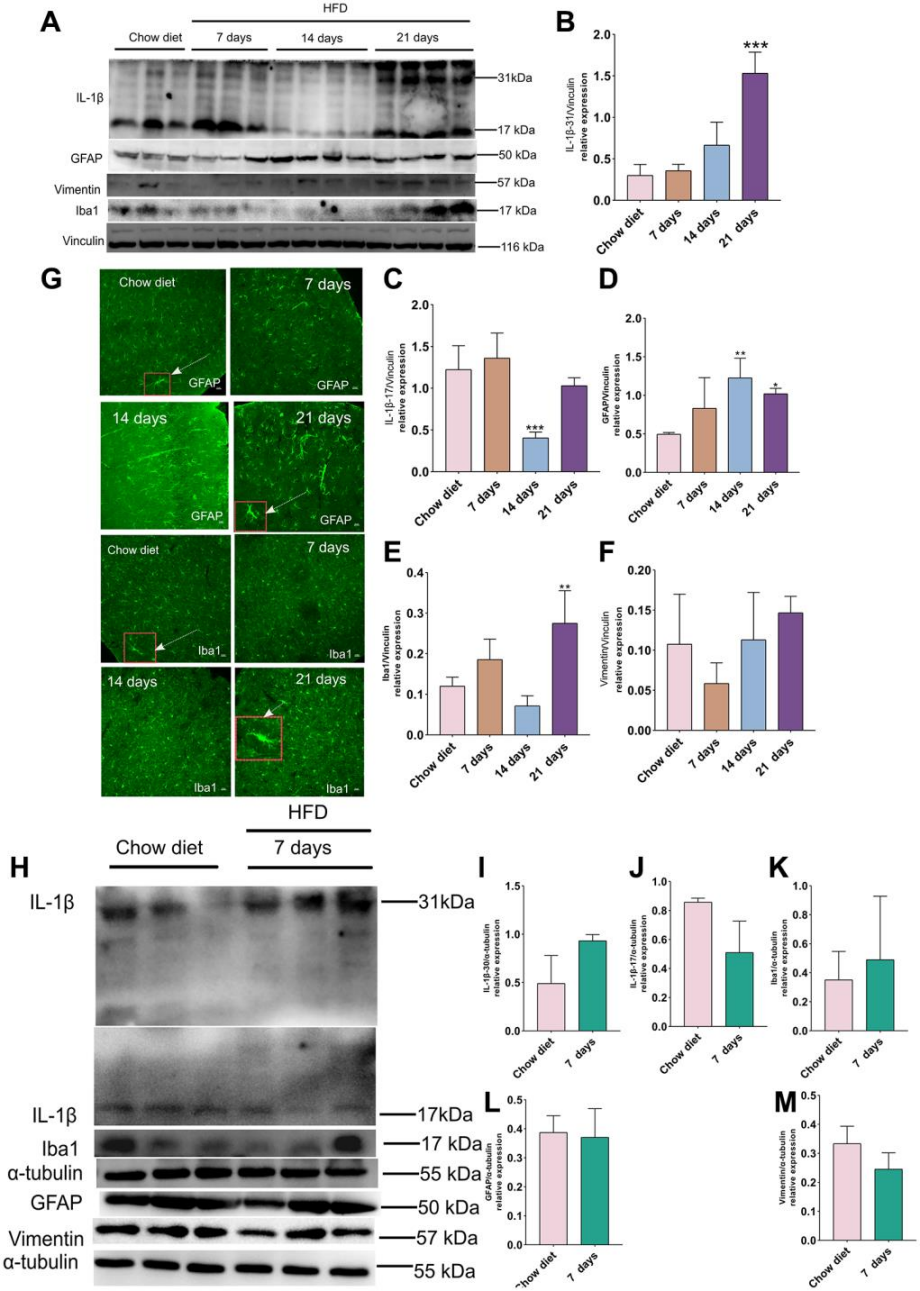
**Effect of HFD on activation of astrocytes and microglia in the mice cortex and cerebellum.** (**A**) Western blots and (**B**–**F**) densitometric analysis for IL-1β 31 kDa (**B**), IL-1β 17 kDa (**C**), GFAP (**D**), Iba-1 (**E**), vimentin (**F**) after HFD in mice cerebral cortex at day 7, day 14 and day 21 (*n* = 3 to 4 per group). (**G**) Immunohistochemistry of GFAP^+^ astrocytes and Iba-1^+^ microglia in cerebral cortex tissue sections. High magnification images of astrocytes and microglia in a larger box outlined by red color indicated by arrows. Magnification, ×40. Scale bars, 10 μm. (**H**) Western blot analysis of cerebellum tissues of mice with HFD and chow diet controls (*n* = 3 per group). (**I**–**M**) Quantification of IL-1β 31 kDa (**I**), IL-1β 17 kDa (**J**), Iba-1 (**K**), GFAP (**L**) and vimentin (**M**).Vinculin and β-actin as a loading control. Values are presented as means ± SD. ^*^P < 0.05 and ^**^P < 0.01 versus chow diet; one-way ANOVA by Tukey’s test for cerebral cortex. Two-tailed Student’s *t* test for cerebellum.

To investigate whether short-term HFD could activate pro-inflammatory actions and glial activation, western blotting and IF staining were conducted with IL-1β (a key player in the regulation of inflammatory processes) [29], Iba-1 (ionized calcium binding adapter protein 1; a hallmark of microglial activity) [30] as well as GFAP and vimentin antibody. As illustrated in Figure 4A, the expression of IL-1β 30 were increased considerably at day 30 compared to chow diet (*p* < 0.001) (Figure 4A–4B) in cerebral cortex. In the meantime, IL-1β 17 remained unchanged after 21 days HFD and showed a decrease at day 14 in these regions (*p* < 0.001) (Figure 4C). On the other hand, the level of GFAP and Iba-1 exhibited evidently higher than chow diet (*p* < 0.05; *p* < 0.01) (Figure 4D–4E). However, vimentin did not display obviously difference at various time course (Figure 4D–4F). Moreover, the IF staining results in Figure 4G also demonstrated hypertrophic astrocytes [31] and hypertrophic microglia [32] delineated by red boxes at day 21 compared to chow diet groups. In addition, consistent with the results from western blot analysis, Quantitation optical density of GFAP^+^ astrocytes (Supplementary Figure 1A) and Iba1^+^ microglia (Supplementary Figure 1B) staining was significantly enhanced at 21 days HFD compared with chow diet group (*p* < 0.01). Therefore, the results indicates that short-term HFD can promote neuroinflammation and glial cell activities in cerebral cortex. Moreover, no significant difference was found in IL-1β 30 and IL-1β 17 between chow diet and HFD mice in cerebellum (Figure 4H–4J). Contrary to cerebral cortex findings at day 21, no changes were observed in GFAP, vimentin and Iba-1 in 7 days HFD-fed mice compared to chow diet mice in cerebellum (Figure 4K–4M). These observations demonstrate that cerebellum may be resistant apoptosis occurrence within 7 days of eating HFD.

### Effects of short-term HFD on Akt/MAPK/NF-κB p65 pathways in cerebral cortex and cerebellum

**Figure 5:**
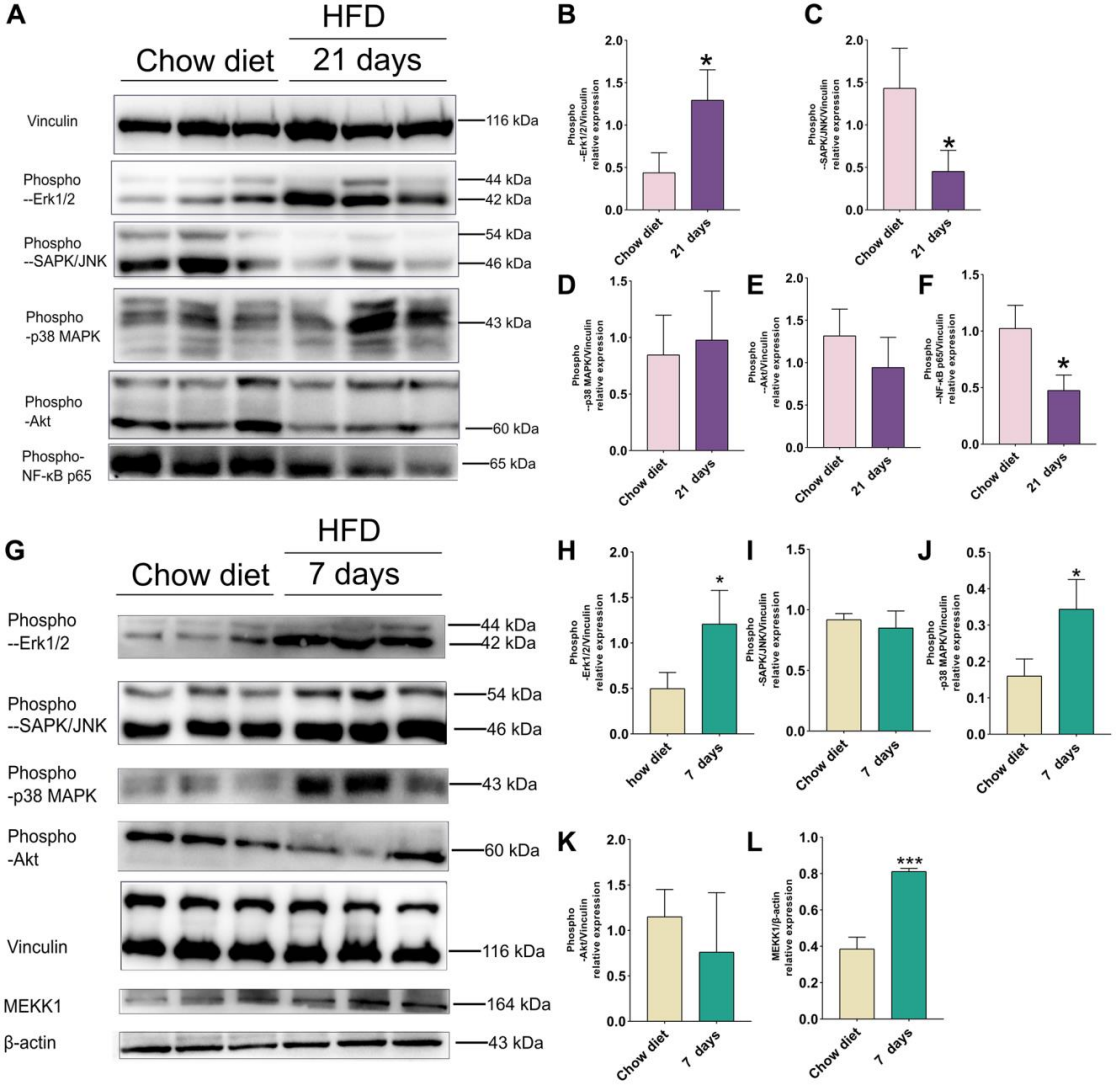
**Effect of HFD on signaling pathways in the mice cortex at 21 days and cerebellum at 7 days.** (**A**) Western blots and (**B**–**F**) Quantitation for phospho-p44/42 MAPK (Erk1/2) (**B**), phospho-SAPK/JNK (**C**), phospho-p38 MAPK(D), phospho-Akt (**E**), phospho-NF-κB p65 (**F**) in the cortex of mice after 21 days HFD (*n* = 3 per group). (**G**) Western blots and (**H**–**L**) Quantitation for phospho-p44/42 MAPK (Erk1/2) (**H**), phospho-SAPK/JNK (**I**), phospho-p38 MAPK (**J**), phospho-Akt (**K**), MEKK1 (**L**) in the cerebellum of mice after 7 days HFD (*n* = 3 per group). Vinculin and β-actin as a loading control. Values are presented as means ± SD. ^*^P < 0.05 and ^**^P < 0.01 versus chow diet; Two-tailed Student’s *t* test.

On the 21st day after HFD in mice cerebral cortex, phosphorylation of ERK1/2 was significantly enhanced compared to chow diet (*p* < 0.05) (Figure 5A–5B). However, JNK and NF-κB p65 phosphorylation demonstrated significant lower than chow diet groups (*p* < 0.05) (Figure 5C, 5F). In addition, no significant differences in phospho-p38 MAPK and phospho-Akt were observed between chow diet and HFD mice (Figure 5D, 5E).

As illustrated in Figure 5G, western blot indicated that activities of phospho- ERK1/2, phospho-p38 MAPK and MEKK1 were increased significantly 1 weeks after HFD-fed mice in cerebellum (*p* < 0.05; *p* < 0.01) (Figure 5H, 5J, 5L). However, no significant results were found in phospho-JNK and phospho-Akt between chow diet and HFD mice (Figure 5I, 5K).

### Cholesterol (a component of HFD) promotes apoptosis of SH-SY5Y Cells after 48h culture and downregulates Akt/MAPK/NF-κB p65 signaling

**Figure 6:**
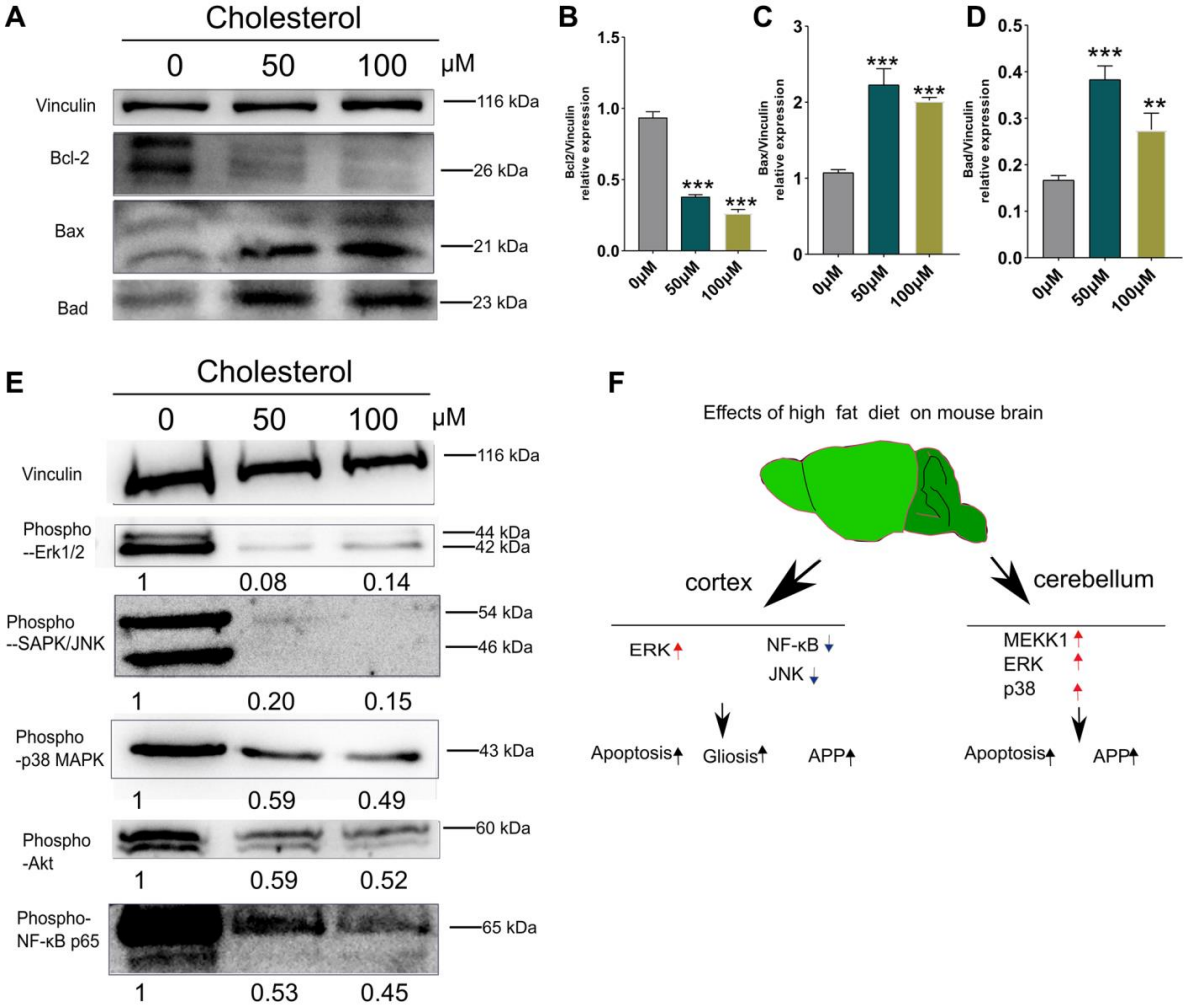
**Changes of apoptosis and signaling pathways induced by cholesterol in SH-SY5Y cells.** SH-SY5Y Cells were treated with cholesterol at indicated concentration (0 μM, 50 μM, and 100 μM) for 48 h (*n* = 3 per group). (**A**) Western blots and (**B**–**D**) Quantitation for Bcl-2 (**B**), Bax (**C**), and Bad (**D**) in the supernatants of SH-SY5Y Cells. (**E**) Western blots of phospho-p44/42 MAPK (Erk1/2), phospho-SAPK/JNK, phospho-p38 MAPK, phospho-Akt, phospho-NF-κB p65 from supernatants of SH-SY5Y Cells after 48 h culture. The levels of phosphorylated signaling-related proteins after treatment with 50 μM, and 100 μM cholesterol were normalized to the levels of the 0μM treatment group. (**F**) Diagram showing the mechanism of how short-term HFD aggravates apoptosis, glial cell activation and APP production in cerebral cortex and cerebellum. Vinculin as a loading control. Values are presented as means ± SD. ^*^P < 0.05 and ^**^P < 0.01 versus chow diet; one-way ANOVA by Tukey’s test. Red arrow indicates up-regulation and Black arrow indicates down-regulation.

As shown in Figure 6A–6E, the stimulation of SH-SY5Y cells with 50 μM and 100 μM cholesterol [33] significantly decreased the level Bcl-2 in associated with evident increased Bax and Bad, compared with control group (0 μM) (*p* < 0.01; *p* < 0.001) (Figure 6B–6D). Interestingly, phospho-ERK1/2, phospho-JNK phospho-p38 MAPK and phospho-Akt were obviously reduced compared to control group (0 μM) (Figure 6E). Altogether, while cholesterol-treated cell models *in vitro* can partially simulate apoptosis in cortex and cerebellum of HFD-fed mice, these results do not represent the changes of stress signaling *in vivo*.

Schematic explanation suggests that activation of ERK1/2 pathways was occurred in mice cortex after 21 days HFD feeding. Meanwhile, ERK1/2, p38 MAPK and MEKK1 signaling were elevated simultaneously in cerebellum at day 7 of HFD-fed mice (Figure 6F).

## DISCUSSION

In this study, we investigated the effects of short-term HFD on APP expression, apoptosis, IL-1β, glial cell activities as well as MAPK, Akt, NF-κB p65 pathways in the mouse cerebral cortex and cerebellum. The results in the C57BL/6J mice demonstrated that intake of HFD for only 7 days resulted in increased APP level in cortex and cerebellum. Furthermore, HFD consumption significantly contributed to apoptosis appearance, IL-1β elevation and gliosis at different time courses from 7 days to 21 days in cerebral cortex region. Meanwhile, HFD intake after 7 days triggered apoptosis increase in cerebellum. Nevertheless, no alterations of gliosis and IL-1β were brought about for cerebellum. Unexpectedly, increased activation of ERK1/2 level was observed both in cerebral cortex and cerebellum in HFD. In addition, *in vivo* studies showed that cholesterol advanced apoptosis of SH-SY5Y cells accompanying reduction activities of Akt/MAPK/NF-κBp65 signaling. Our data show a possible mechanism in the pathophysiology of apoptosis induction and glial cell activities associated with cerebral cortex and cerebellum dysfunctions under HFD conditions. Taken together, we demonstrated that short-term HFD consumption differentially contributes to apoptosis, neuroinflammation and APP expression in brain accompanying increased activity of ERK1/2 and p38 MAPK, which appears to be related to brain regions-dependent and time-dependent changes.

Interestingly, some reports indicate that dietary administration of HFD in APP transgenic mice do not alter the production of APP [34, 35], while other finding demonstrates that HFD promotes APP expression [36]. On the other hand, other reports claim that APP production was increased after HFD for 4 weeks in normal adult rats [37] and no changes for APP mRNA level after short-term HFD in 4 weeks-old ordinary mice [13]. In the present study, however, our results showed that increased APP production continued to exist from day 7 to day 21 after transitory HFD. Similar to studies in APP after, whether HFD affect the level of BACE1 expression remains controversial. Miren Ettchetoa et al. reported that BACE1 protein level was not influenced by HFD in APP/PS1 mice [36]. On the contrary, high fat/cholesterol induced significantly increased expression of BACE1 in C57BL/6 mice [9]. Our results suggested that fluctuating changes of BACE1 were seen in cerebral cortex, and these changes were significant in day 7 when compared to chow diet group. But there was no changes in BACE1 level in cerebellum. On the other hand, APP is cleaved by BACE1 (β-site amyloid precursor protein cleaving enzyme-1), contributing to Aβ aggregation. However, we did not detect Aβ (β-Amyloid) formation in the supernatants by western blots (data not shown). Thus, we speculate that temporary HFD leading to increased APP may be related to enhanced apoptotic process [38, 39], but not Alzheimer’s development. Together, although the exact reason for the discrepancy between their results and ours remain unknown, we assume that it may be due to the changes in detection methods or experimental design.

In line with previous research, HFD administration can result in an increase of microglia, astrocytes and neuroinflammation in brain [2, 10, 12, 40]. We found that GFAP (Glial fibrillary acidic protein; markers for the activation of astrocytes) was increased significantly at day 14 and day 21after HFD. However, vimentin level (type III intermediate filament protein; a marker for astrocytes) remained unchanged during short-term HFD. It is possible that vimentin is also present in other cell types of brain [41, 42]. In addition, Iba-1 level also was raised after 21 days HFD. Furthermore, IL-1β 30 and IL-1β 17 expression increased significantly at day 21 and day 14 and did not change significantly in the other time points compared to chow diet in cerebral cortex. Thus, we reasoned that increased astrocytes and microglia may contribute to IL-1β production [43]. HFD induces neuroinflammation in the cerebral cortex through microglia and astrocytes activation. However, unchanged of GFAP, vimentin and Iba-1 level were observed in cerebellum at day 7 after HFD. This phenomenon in our study is consistent with IL-1β results in cerebellum that did not show significant difference between HFD and chow diet. Finally, our results clearly demonstrate region-dependent and time-dependent changes in specific brain regions in response to the HFD.

Currently, many reports have indicated that MAPK signaling are involved in response to metabolic stresses changes of extracellular or intracellular signals, such as glucose, circulating free fatty acids (FFAs) and intake HFD, which affect cell and organ metabolism [17, 44]. In agreement with previous research reported that an increase in the activity of ERK1/2 after long-term HFD (20weeks) in hippocampus [19], our studies also revealed that 21 days HFD feeding increased the level of phospho-ERK1/2 significantly in cerebral cortex. However, other evidence have shown that after 16weeks of HFD feeding decrease ERK activity in the entire brain [20]. Recently, research indicates that stimulation of ERK1/2 can cause neural cell death [22, 45, 46]. Thus, our results suggest that upregulation of ERK1/2 activity in short-term HFD may contribute to apoptosis happening, gliosis and increased APP level. In addition, many new findings in mice and humans suggest that JNK activation might induce insulin resistance and lead to obesity [47, 48]. Meanwhile, NF-κB inflammatory regulation signaling pathway is up-regulated in the brain tissue of 14weeks or 16weeks HFD-feeding mice [20, 49] as well NF-κB inflammatory signaling is unchanged in subcutaneous white adipose tissue after short-term high-fat overfeeding [16]. On the other hand, our results showed that after 7 days or 21 days consumption of HFD led to a significant decrease in phospho-NF-κB p65 and phospho-JNK simultaneously. Thus, we reasoned that the differences of results are mainly due to the experimental duration and tissue location.

Interestingly, we also observed the increased activities of MEKK1/ERK1/2 in cerebellum. In consistent with Liu et al. have reported phospho- p38MAPK increase after extending HFD feeding [20, 49], our results demonstrated that p38MAPK activity was enhanced significantly after 7 days HFD. Some findings presented in this Review show that activation of p38MAPK lead to insulin resistance, obesity and metabolic diseases [48]. Therefore, activation of MEKK1/ERK1/2 and p38MAPK aggravated the vulnerability of cerebellum to 7 days HFD feeding.

However, the limitations of current study should be stated. First, because similar results have been described in some literature [12, 13, 19], we did not explore the changes of metabolic parameters, such as glucose, cholesterol, insulin and triglyceride levels in blood, which may reflect changes of metabolic variables in short-term HFD-fed mice. Nevertheless, more recent studies suggest that cholesterol and triglyceride can induce neuroinflammation [40, 50]. Therefore, it is worth investigating the associations between metabolism and apoptosis in brain over time following short-term HFD. Second, gender and biological sex affect the pathogenesis of metabolic disorders, such as diabetes, obesity, insulin resistance and hyperglycemia [51, 52]. We used male mice only for the experiment. Therefore, whether there is an association between the effects of short-term HFD on brain and mice gender should be evaluated. Third, blood–brain barrier (BBB) remains intact after one-week or 12 month exposure to high-fat diet [53, 54]. Although we observed the up-regulation of ERK1/2 protein in brain of HFD mice, ERK1/2 inhibitors cannot be used to prove whether decreased ERK1/2 will reverse apoptosis in brain after short-term HFD. Because no sufficient evidence shows that ERK1/2 inhibitors will cross the BBB. We believe that nanoparticle technology may contribute to facilitating inhibitors delivery to transverse the BBB in the future.

In conclusion, we discovered that short periods (7 days, 14 days and 21 days) consumption of HFD caused some alterations of APP protein, apoptosis, glial cell, IL-1β and MAPK signaling in cerebral cortex and cerebellum of young mice. Interestingly, apoptotic molecules changes have started from the first week. In addition, it is necessary to identify the exact roles of ERK1/2 and APP up-regulation in different regions of brain after short-term HFD.

## Supplementary Materials

Supplementary Figure 1

## References

[1] Friedman JM. Obesity: Causes and control of excess body fat. Nature. 2009; 459:340–42. 10.1038/459340a 19458707

[2] Keshk WA, Ibrahim MA, Shalaby SM, Zalat ZA, Elseady WS. Redox status, inflammation, necroptosis and inflammasome as indispensable contributors to high fat diet (HFD)-induced neurodegeneration; Effect of N-acetylcysteine (NAC). Arch Biochem Biophys. 2020; 680:108227. 10.1016/j.abb.2019.108227 31838118

[3] Laaksonen DE, Niskanen L, Lakka HM, Lakka TA, Uusitupa M. Epidemiology and treatment of the metabolic syndrome. Ann Med. 2004; 36:332–46. 10.1080/07853890410031849 15478308

[4] Décarie-Spain L, Sharma S, Hryhorczuk C, Issa-Garcia V, Barker PA, Arbour N, Alquier T, Fulton S. Nucleus accumbens inflammation mediates anxiodepressive behavior and compulsive sucrose seeking elicited by saturated dietary fat. Mol Metab. 2018; 10:1–13. 10.1016/j.molmet.2018.01.018 29454579 PMC5985233

[5] de Aquino CC, Leitão RA, Oliveira Alves LA, Coelho-Santos V, Guerrant RL, Ribeiro CF, Malva JO, Silva AP, Oriá RB. Effect of Hypoproteic and High-Fat Diets on Hippocampal Blood-Brain Barrier Permeability and Oxidative Stress. Front Nutr. 2019; 5:131. 10.3389/fnut.2018.00131 30687711 PMC6333637

[6] Park HS, Cho HS, Kim TW. Physical exercise promotes memory capability by enhancing hippocampal mitochondrial functions and inhibiting apoptosis in obesity-induced insulin resistance by high fat diet. Metab Brain Dis. 2018; 33:283–92. 10.1007/s11011-017-0160-8 29185193

[7] Qin S, Sun D, Zhang C, Tang Y, Zhou F, Zheng K, Tang R, Zheng Y. Downregulation of sonic hedgehog signaling in the hippocampus leads to neuronal apoptosis in high-fat diet-fed mice. Behav Brain Res. 2019; 367:91–100. 10.1016/j.bbr.2019.03.055 30940514

[8] Lu J, Wu DM, Zheng ZH, Zheng YL, Hu B, Zhang ZF. Troxerutin protects against high cholesterol-induced cognitive deficits in mice. Brain. 2011; 134:783–97. 10.1093/brain/awq376 21252113

[9] Hou J, Jeon B, Baek J, Yun Y, Kim D, Chang B, Kim S, Kim S. High fat diet-induced brain damaging effects through autophagy-mediated senescence, inflammation and apoptosis mitigated by ginsenoside F1-enhanced mixture. Journal of Ginseng Research. 2021. [Epub ahead of print]. 10.1016/j.jgr.2021.04.002 PMC8753566 35058728

[10] Saiyasit N, Chunchai T, Apaijai N, Pratchayasakul W, Sripetchwandee J, Chattipakorn N, Chattipakorn SC. Chronic high-fat diet consumption induces an alteration in plasma/brain neurotensin signaling, metabolic disturbance, systemic inflammation/oxidative stress, brain apoptosis, and dendritic spine loss. Neuropeptides. 2020; 82:102047. 10.1016/j.npep.2020.102047 32327191

[11] Holloway CJ, Cochlin LE, Emmanuel Y, Murray A, Codreanu I, Edwards LM, Szmigielski C, Tyler DJ, Knight NS, Saxby BK, Lambert B, Thompson C, Neubauer S, Clarke K. A high-fat diet impairs cardiac high-energy phosphate metabolism and cognitive function in healthy human subjects. Am J Clin Nutr. 2011; 93:748–55. 10.3945/ajcn.110.002758 21270386

[12] Sobesky JL, D'Angelo HM, Weber MD, Anderson ND, Frank MG, Watkins LR, Maier SF, Barrientos RM. Glucocorticoids Mediate Short-Term High-Fat Diet Induction of Neuroinflammatory Priming, the NLRP3 Inflammasome, and the Danger Signal HMGB1. eNeuro. 2016; 3:ENEURO.0113-16.2016. 10.1523/ENEURO.0113-16.2016 27595136 PMC5004086

[13] Nakandakari SC, Muñoz VR, Kuga GK, Gaspar RC, Sant'Ana MR, Pavan ICB, da Silva LGS, Morelli AP, Simabuco FM, da Silva ASR, de Moura LP, Ropelle ER, Cintra DE, Pauli JR. Short-term high-fat diet modulates several inflammatory, ER stress, and apoptosis markers in the hippocampus of young mice. Brain Behav Immun. 2019; 79:284–93. 10.1016/j.bbi.2019.02.016 30797044

[14] Spencer SJ, D'Angelo H, Soch A, Watkins LR, Maier SF, Barrientos RM. High-fat diet and aging interact to produce neuroinflammation and impair hippocampal- and amygdalar-dependent memory. Neurobiol Aging. 2017; 58:88–101. 10.1016/j.neurobiolaging.2017.06.014 28719855 PMC5581696

[15] Beilharz JE, Maniam J, Morris MJ. Short-term exposure to a diet high in fat and sugar, or liquid sugar, selectively impairs hippocampal-dependent memory, with differential impacts on inflammation. Behav Brain Res. 2016; 306:1–7. 10.1016/j.bbr.2016.03.018 26970578

[16] Dewhurst-Trigg R, Wadley AJ, Woods RM, Sherar LB, Bishop NC, Hulston CJ, Markey O. Short-term High-fat Overfeeding Does Not Induce NF-κB Inflammatory Signaling in Subcutaneous White Adipose Tissue. J Clin Endocrinol Metab. 2020; 105:dgaa158. 10.1210/clinem/dgaa158 32232380

[17] Wu JJ, Roth RJ, Anderson EJ, Hong EG, Lee MK, Choi CS, Neufer PD, Shulman GI, Kim JK, Bennett AM. Mice lacking MAP kinase phosphatase-1 have enhanced MAP kinase activity and resistance to diet-induced obesity. Cell Metab. 2006; 4:61–73. 10.1016/j.cmet.2006.05.010 16814733

[18] Czarzasta K, Koperski L, Segiet A, Janiszewski M, Kuch M, Gornicka B, Cudnoch-Jedrzejewska A. The role of high fat diet in the regulation of MAP kinases activity in left ventricular fibrosis. Acta Histochem. 2019; 121:303–10. 10.1016/j.acthis.2019.01.010 30733042

[19] Abbasnejad Z, Nasseri B, Zardooz H, Ghasemi R. Time-course study of high fat diet induced alterations in spatial memory, hippocampal JNK, P38, ERK and Akt activity. Metab Brain Dis. 2019; 34:659–73. 10.1007/s11011-018-0369-1 30552557

[20] Mi Y, Qi G, Fan R, Qiao Q, Sun Y, Gao Y, Liu X. EGCG ameliorates high-fat- and high-fructose-induced cognitive defects by regulating the IRS/AKT and ERK/CREB/BDNF signaling pathways in the CNS. FASEB J. 2017; 31:4998–5011. 10.1096/fj.201700400RR 28739640

[21] Nishimoto S, Nishida E. MAPK signalling: ERK5 versus ERK1/2. EMBO Rep. 2006; 7:782–86. 10.1038/sj.embor.7400755 16880823 PMC1525153

[22] Li F, Omori N, Sato K, Jin G, Nagano I, Manabe Y, Shoji M, Abe K. Coordinate expression of survival p-ERK and proapoptotic cytochrome c signals in rat brain neurons after transient MCAO. Brain Res. 2002; 958:83–88. 10.1016/s0006-8993(02)03465-0 12468032

[23] Thornton TM, Rincon M. Non-classical p38 map kinase functions: cell cycle checkpoints and survival. Int J Biol Sci. 2009; 5:44–51. 10.7150/ijbs.5.44 19159010 PMC2610339

[24] Xu CJ, Wang JL, Jing-Pan, Min-Liao. Tph2 Genetic Ablation Contributes to Senile Plaque Load and Astrogliosis in APP/PS1 Mice. Curr Alzheimer Res. 2019; 16:219–32. 10.2174/1567205016666190301110110 30827242

[25] Xu CJ, Xu L, Huang LD, Li Y, Yu PP, Hang Q, Xu XM, Lu PH. Combined NgR vaccination and neural stem cell transplantation promote functional recovery after spinal cord injury in adult rats. Neuropathol Appl Neurobiol. 2011; 37:135–55. 10.1111/j.1365-2990.2010.01117.x 20819171

[26] Wang JL, Chen WG, Zhang JJ, Xu CJ. Nogo-A-Δ20/EphA4 interaction antagonizes apoptosis of neural stem cells by integrating p38 and JNK MAPK signaling. J Mol Histol. 2021; 52:521–37. 10.1007/s10735-021-09960-6 33555537

[27] Wang JL, Zhao L, Li MQ, Chen WG, Xu CJ. A sensitive and reversible staining of proteins on blot membranes. Anal Biochem. 2020; 592:113579. 10.1016/j.ab.2020.113579 31926891

[28] González-Gualda E, Baker AG, Fruk L, Muñoz-Espín D. A guide to assessing cellular senescence in vitro and in vivo. FEBS J. 2021; 288:56–80. 10.1111/febs.15570 32961620

[29] Gabay C, Lamacchia C, Palmer G. IL-1 pathways in inflammation and human diseases. Nat Rev Rheumatol. 2010; 6:232–41. 10.1038/nrrheum.2010.4 20177398

[30] Busquets O, Ettcheto M, Pallàs M, Beas-Zarate C, Verdaguer E, Auladell C, Folch J, Camins A. Long-term exposition to a high fat diet favors the appearance of β-amyloid depositions in the brain of C57BL/6J mice. A potential model of sporadic Alzheimer's disease. Mech Ageing Dev. 2017; 162:38–45. 10.1016/j.mad.2016.11.002 27863851

[31] Wilhelmsson U, Bushong EA, Price DL, Smarr BL, Phung V, Terada M, Ellisman MH, Pekny M. Redefining the concept of reactive astrocytes as cells that remain within their unique domains upon reaction to injury. Proc Natl Acad Sci U S A. 2006; 103:17513–18. 10.1073/pnas.0602841103 17090684 PMC1859960

[32] Martini AC, Helman AM, McCarty KL, Lott IT, Doran E, Schmitt FA, Head E. Distribution of microglial phenotypes as a function of age and Alzheimer's disease neuropathology in the brains of people with Down syndrome. Alzheimers Dement (Amst). 2020; 12:e12113. 10.1002/dad2.12113 33088896 PMC7560512

[33] Avila-Muñoz E, Arias C. Cholesterol-induced astrocyte activation is associated with increased amyloid precursor protein expression and processing. Glia. 2015; 63:2010–22. 10.1002/glia.22874 26096015

[34] Julien C, Tremblay C, Phivilay A, Berthiaume L, Emond V, Julien P, Calon F. High-fat diet aggravates amyloid-beta and tau pathologies in the 3xTg-AD mouse model. Neurobiol Aging. 2010; 31:1516–31. 10.1016/j.neurobiolaging.2008.08.022 18926603

[35] Maesako M, Uemura K, Kubota M, Kuzuya A, Sasaki K, Asada M, Watanabe K, Hayashida N, Ihara M, Ito H, Shimohama S, Kihara T, Kinoshita A. Environmental enrichment ameliorated high-fat diet-induced Aβ deposition and memory deficit in APP transgenic mice. Neurobiol Aging. 2012; 33:1011.e11–23. 10.1016/j.neurobiolaging.2011.10.028 22197104

[36] Ettcheto M, Petrov D, Pedrós I, Alva N, Carbonell T, Beas-Zarate C, Pallas M, Auladell C, Folch J, Camins A. Evaluation of Neuropathological Effects of a High-Fat Diet in a Presymptomatic Alzheimer's Disease Stage in APP/PS1 Mice. J Alzheimers Dis. 2016; 54:233–51. 10.3233/JAD-160150 27567882

[37] Selvi Y, Gergerlioglu HS, Akbaba N, Oz M, Kandeger A, Demir EA, Yerlikaya FH, Nurullahoglu-Atalik KE. Impact of enriched environment on production of tau, amyloid precursor protein and, amyloid-β peptide in high-fat and high-sucrose-fed rats. Acta Neuropsychiatr. 2017; 29:291–98. 10.1017/neu.2016.63 27923413

[38] Kögel D, Concannon CG, Müller T, König H, Bonner C, Poeschel S, Chang S, Egensperger R, Prehn JH. The APP intracellular domain (AICD) potentiates ER stress-induced apoptosis. Neurobiol Aging. 2012; 33:2200–09. 10.1016/j.neurobiolaging.2011.06.012 21803450

[39] Chen YZ. APP induces neuronal apoptosis through APP-BP1-mediated downregulation of beta-catenin. Apoptosis. 2004; 9:415–22. 10.1023/B:APPT.0000031447.05354.9f 15192323

[40] Thirumangalakudi L, Prakasam A, Zhang R, Bimonte-Nelson H, Sambamurti K, Kindy MS, Bhat NR. High cholesterol-induced neuroinflammation and amyloid precursor protein processing correlate with loss of working memory in mice. J Neurochem. 2008; 106:475–85. 10.1111/j.1471-4159.2008.05415.x 18410513 PMC3897170

[41] Levin EC, Acharya NK, Sedeyn JC, Venkataraman V, D'Andrea MR, Wang HY, Nagele RG. Neuronal expression of vimentin in the Alzheimer's disease brain may be part of a generalized dendritic damage-response mechanism. Brain Res. 2009; 1298:194–207. 10.1016/j.brainres.2009.08.072 19728994

[42] Jiang SX, Slinn J, Aylsworth A, Hou ST. Vimentin participates in microglia activation and neurotoxicity in cerebral ischemia. J Neurochem. 2012; 122:764–74. 10.1111/j.1471-4159.2012.07823.x 22681613

[43] Shaftel SS, Griffin WS, O'Banion MK. The role of interleukin-1 in neuroinflammation and Alzheimer disease: an evolving perspective. J Neuroinflammation. 2008; 5:7. 10.1186/1742-2094-5-7 18302763 PMC2335091

[44] Gehart H, Kumpf S, Ittner A, Ricci R. MAPK signalling in cellular metabolism: stress or wellness? EMBO Rep. 2010; 11:834–40. 10.1038/embor.2010.160 20930846 PMC2966959

[45] Blázquez C, Galve-Roperh I, Guzmán M. De novo-synthesized ceramide signals apoptosis in astrocytes via extracellular signal-regulated kinase. FASEB J. 2000; 14:2315–22. 10.1096/fj.00-0122com 11053253

[46] Murray B, Alessandrini A, Cole AJ, Yee AG, Furshpan EJ. Inhibition of the p44/42 MAP kinase pathway protects hippocampal neurons in a cell-culture model of seizure activity. Proc Natl Acad Sci U S A. 1998; 95:11975–80. 10.1073/pnas.95.20.11975 9751775 PMC21750

[47] Sabio G, Davis RJ. cJun NH2-terminal kinase 1 (JNK1): roles in metabolic regulation of insulin resistance. Trends Biochem Sci. 2010; 35:490–96. 10.1016/j.tibs.2010.04.004 20452774 PMC2975251

[48] Nikolic I, Leiva M, Sabio G. The role of stress kinases in metabolic disease. Nat Rev Endocrinol. 2020; 16:697–716. 10.1038/s41574-020-00418-5 33067545

[49] Mulati A, Ma S, Zhang H, Ren B, Zhao B, Wang L, Liu X, Zhao T, Kamanova S, Sair AT, Liu Z, Liu X. Sea-Buckthorn Flavonoids Alleviate High-Fat and High-Fructose Diet-Induced Cognitive Impairment by Inhibiting Insulin Resistance and Neuroinflammation. J Agric Food Chem. 2020; 68:5835–46. 10.1021/acs.jafc.0c00876 32363873

[50] Lee LL, Aung HH, Wilson DW, Anderson SE, Rutledge JC, Rutkowsky JM. Triglyceride-rich lipoprotein lipolysis products increase blood-brain barrier transfer coefficient and induce astrocyte lipid droplets and cell stress. Am J Physiol Cell Physiol. 2017; 312:C500–16. 10.1152/ajpcell.00120.2016 28077357 PMC5407020

[51] Palmisano BT, Zhu L, Eckel RH, Stafford JM. Sex differences in lipid and lipoprotein metabolism. Mol Metab. 2018; 15:45–55. 10.1016/j.molmet.2018.05.008 29858147 PMC6066747

[52] Tramunt B, Smati S, Grandgeorge N, Lenfant F, Arnal JF, Montagner A, Gourdy P. Sex differences in metabolic regulation and diabetes susceptibility. Diabetologia. 2020; 63:453–61. 10.1007/s00125-019-05040-3 31754750 PMC6997275

[53] Elhaik Goldman S, Goez D, Last D, Naor S, Liraz Zaltsman S, Sharvit-Ginon I, Atrakchi-Baranes D, Shemesh C, Twitto-Greenberg R, Tsach S, Lotan R, Leikin-Frenkel A, Shish A, et al. High-fat diet protects the blood-brain barrier in an Alzheimer's disease mouse model. Aging Cell. 2018; 17:e12818. 10.1111/acel.12818 30079520 PMC6156545

[54] Rijnsburger M, Unmehopa UA, Eggels L, Serlie MJ, la Fleur SE. One-week exposure to a free-choice high-fat high-sugar diet does not disrupt blood-brain barrier permeability in fed or overnight fasted rats. Nutr Neurosci. 2019; 22:541–50. 10.1080/1028415X.2017.1418727 29284375

