# Supplementary material for: Short-term high-fat diet favors the appearances of apoptosis and gliosis by activation of ERK1/2/p38MAPK pathways in brain
Source: Aging (Albany NY). 2021 Oct 7;13(19):23133–48. doi: 10.18632/aging.203607 (PMC8544319; doi:10.18632/aging.203607)
Supplement: Supplementary Figure 1 [file aging-13-203607-s001.pdf]

SUPPLEMENTARY FIGURE

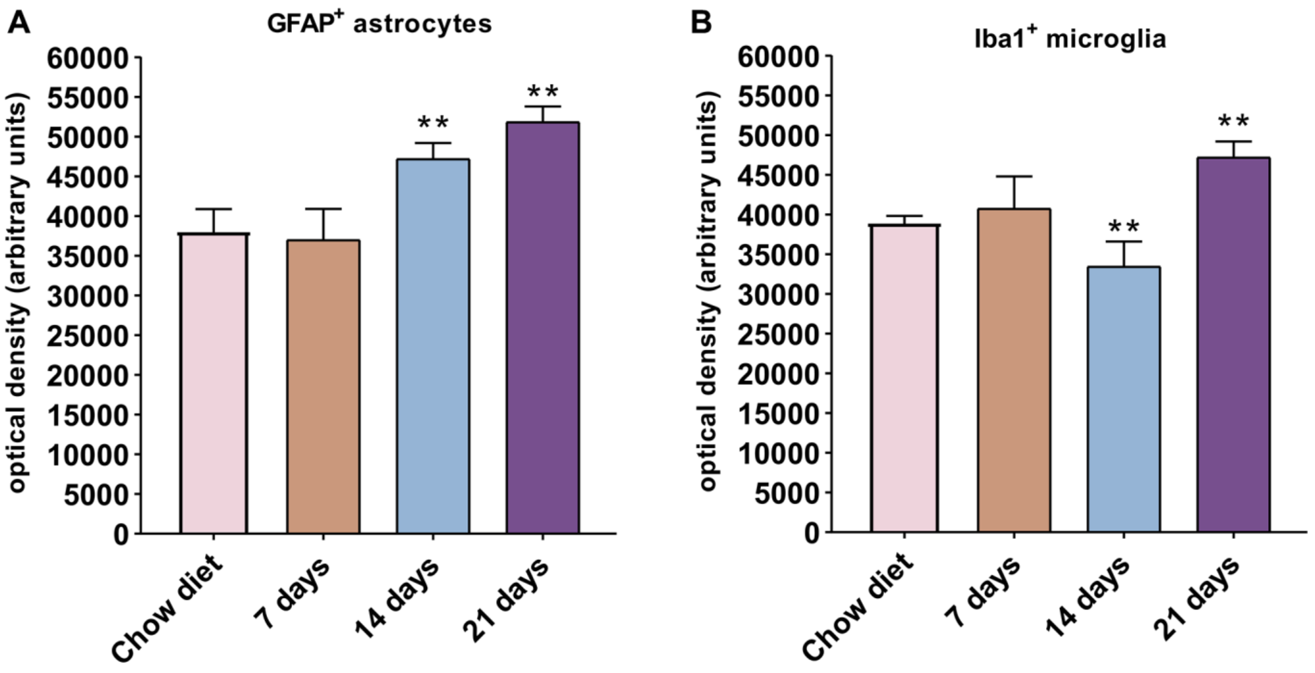

Supplementary Figure 1. Quantitation optical density of GFAP+ astrocytes (**A**) and Iba1+ microglia (**B**) staining of mice fed chow diet and high fat diet in different time points. The images (2–3 sections per animal) were captured and quantified using the NIH Image J software package. $^{*}p < 0.01$ versus the chow diet fed control group.
